# The Quality Evaluation of Postharvest Strawberries Stored in Nano-Ag Packages at Refrigeration Temperature

**DOI:** 10.3390/polym10080894

**Published:** 2018-08-09

**Authors:** Cheng Zhang, Wenhui Li, Bifen Zhu, Haiyan Chen, Hai Chi, Lin Li, Yuyue Qin, Jing Xue

**Affiliations:** 1 Institute of Yunnan Food Safety, Kunming University of Science and Technology, Kunming 650500, China; 13136640259@163.com (C.Z.); 15559823733@163.com (W.L.); 18469189957@163.com (B.Z.); seacome@163.com (H.C.); 18468273140@163.com (H.C.); 2 College of Food Sciences and Engineering, South China University of Technology, Guangzhou 510640, China; felinli@scut.edu.cn; 3 State Key Laboratory of Oral Diseases, Sichuan University, Chengdu 610041, China

**Keywords:** strawberry, nano-Ag packaging, storage, quality change

## Abstract

Different percentages (0%, 1%, 5%, and 10%) of nano-Ag particles were added to polylactic acid (PLA) to make an active nanocomposite packaging film. Strawberries were packaged by the nanocomposite films and stored at 4 ± 1 °C for 10 days. The freshness of strawberries was assessed by regularly measuring the physicochemical properties of the strawberries in each packaging film. The difference in the freshness of strawberries was evaluated by determining the following parameter changes: weight loss, hardness, soluble solids, titratable acid, color, vitamin C, total phenol, free radical scavenging activity, peroxidase activity, and sensory evaluation. The results revealed that the active nanocomposite packaging film has better preservation effect when compared with pure PLA film. Its preservation effect is mainly reflected in the more effective reduction of vitamin C loss, delaying the decline of total phenols and 1-Diphenyl-2-picrylhydrazyl (DPPH) in strawberries. It also showed better physical properties. The results showed that the PLA nanocomposite packaging film could effectively preserve freshness of strawberries.

## 1. Introduction

Strawberry (*Fragaria x ananassa* Duch.) is a perennial herb of the genus *Rosaceae*. Strawberry has a unique scent and is juicy. It is rich in vitamins, carotene, anthocyanins, and other nutrients. It is known as the Queen of fruits and widely welcomed by consumers. However, due to its delicate skin, it can easily cause surface crushing in picking and transportation projects. After picking, the strawberry is vigorously breathing and can easily cause spoilage of the fruit [1]. Strawberry is a non-climacteric fruit that can be harvested at maturity to obtain the best taste of the fruit. However, the best tasting period for strawberry is very short, so how to keep strawberries fresh is always the focus of research work. At present, the methods for keeping fresh strawberry mainly include low-temperature refrigeration and fresh-keeping technology [2], modified atmosphere packaging storage technology [3], UV shortwave ultraviolet radiation technology [4], chemical preservation [5] and so on. However, most of these treatments are expensive, time consuming, and may even damage the appearance of the strawberry.

Polylactic acid (PLA) is a biodegradable material and can be regenerated by bacterial fermentation through corn or sugar beets. Due to its low cost of acquisition and biodegradability, it has excellent biocompatibility. PLA is widely studied and used in packaging materials [6]. In addition, PLA has been approved by the US Food and Drug Administration (FDA) and European food regulations for use in food packaging. Thus, it is safe to contact with foodstuff as a packaging material. However, pure PLA is inferior to traditional petroleum-based packaging materials in many aspects such as toughness and thermal properties [7]. In general, pure PLA is polymerized or blended with other biologically active ingredients to improve the desired properties of PLA, such as nano-TiO_2_ [8], nano-Ag, nano-SiO_2_ [9], and nano-ZnO [10].

Nanotechnology is a high-tech science and technology that has been rapidly developed in recent years. It has great market prospects and application value. The addition of nanoparticles not only improves the physical properties of the material such as flexibility, light transmission, and plasticity. It has effective antifungal, microbiological and antiviral activity [11]. Studies have shown that Ag particles have antibacterial effects [12]. Therefore, nano-Ag particles have been widely used in food packaging, textiles, water filtration, and healthcare [13]. Li et al. modified active films by adding nano-Ag into LDPE films and applied them to the preservation of juices. The results show that nano-Ag composite packaging films have greater anti-microbial effects [14]. Therefore, this study embedded nano-Ag particles into PLA to investigate the effect of nano-Ag packaging films on the quality of strawberries stored at 4 ± 1 °C. Then, the effect of nano-Ag PLA-based packaging films on fresh-keeping of strawberry was discussed.

## 2. Materials and Method

### 2.1. Materials

PLA (*M*_w_ = 280 kDa, *M*_w_/*M*_n_ = 1.98) used in this experiment was obtained from Natureworks LLC (Blair, NE, USA). Acetyl tributyl citrate (ATBC) was purchased from Shanghai Macklin Biochemical Co., Ltd. (Shanghai, China). Nano-Ag was purchased from Wanjing New Material Co., Ltd. (Hangzhou, China). Dichloromethane, methanol, NaOH, hydrogen peroxide, Na_2_CO_3_ were obtained from Chengdu Kelong Chemical Co., Ltd. (Chengdu, China). DPPH, Guaiacol were purchased from Sigma (St. Louis, MO, USA). All the reagent were analytical reagent. Texture analyzer (Texture Exponent 32, Stable Micro Systems Ltd., London, UK), Colorimeter (WSC-S; Shanghai Precision Instrument Co., Ltd., Shanghai, China), Digital Refractometer (MZB 92, Shanghai Miqingke Industrial Testing Co., Ltd., Shanghai, China), PH meter (Merck, Barcelona, Spain), spectrophotometry (UV-1800, Mapada Instruments Co., Ltd., Shanghai, China), Centrifuged (TGL-16M, Xiangyi Centrifuge instruments Co., Ltd., Shanghai, China).

### 2.2. Preparation of Film and Sample

The PLA-based nanocomposite films were prepared using the solvent evaporation method according to Qin et al. [15] with some modifications. PLA containing 1 wt % of ATBC plasticizer and different percent of nano-Ag (0, 1, 5, and 10 wt % of 2 g PLA) were dissolved in 50 mL of dichloromethane. The solution was stirred by magnetic stirrer at room temperature for about 10 h. When the solution was completely blended, it was poured onto a 200 mm × 200 mm polytetrafluoroethylene plate and left to stand overnight in the well-ventilated place. Nano-Ag was incorporated into PLA as 0, 1, 5, and 10 wt % loading, and named as PLA, PLA/Ag1%, PLA/Ag5%, and PLA/Ag10%.

Fresh strawberry was harvested from Kunming strawberry greenhouse. About 70–80% ripeness (approximately 70–80% of strawberry retained the red color), same size, no pests, and no mechanical damage extrusion strawberries were selected as the test material. The strawberries were transported to the laboratory immediately after being harvested. Strawberry samples picked from the greenhouse were simply cleaned of soil on the surface and put directly into the bag. Note that water was not used to wash the surface of the strawberry; the soil and other dirt was simply shaken off. Alcohol was applied to both sides of the films and then they were placed on an aseptic table. The films were irradiated with UV light for 15 min to ensure film hygiene as well as to not affect the accuracy of subsequent microbial experiments. Ten fresh strawberries were randomly selected to per type of material package and stored at 4 ± 1 °C. A sample was taken every two days to determine the indicators during 10 days (on Days 0, 2, 4, 6, 8, and 10).

#### Refrigerated Storage

Strawberries were packaged with five kinds of active films (PLA, PLA/Ag1%, PLA/Ag5%, and PLA/Ag10%). Following treatment, strawberries were stored at 4 ± 1 °C for 10 days. The effectiveness of the treatments was evaluated by determining quality changes every two days. Then, combined with sensory evaluation, the acceptability of strawberry preservation was obtained.

### 2.3. Weight Loss (LS)

Three bags were selected randomly from the four active packages to determine the weight every two days, and compare the weight difference with the original fresh weight on the first day. Gravimetry was used to measure the weight loss, expressed as the percentage of the original weight. Equation (1) can be used to express weight loss:

(1) $$\mathrm{Weight}\;\mathrm{Loss}(\%)\;=\;\frac{M_{0}-M_{1}}{M_{0}}\;\times \;100$$

*M*_0_ is the fresh weight of fruit on the first day, and *M*_1_ is the measured weight on each sampling day [16].

### 2.4. Firmness Measurement

The firmness of the strawberry was determined using a texture analyzer (Texture Exponent 32, Stable Micro Systems Ltd., London, UK) equipped with 2 mm diameter cylindrical probe. Four different locations were used to measure firmness around the equatorial region on each fruit. The penetration depth of the probe into the sample was 5 mm and the crosshead speed of the texture analyzer as 2 mm/s. From the force vs. time curve, the maximum force firmness was expressed in N·cm^−2^.

### 2.5. Surface Color

A colorimeter (WSC-S; Shanghai Precision Instrument Co., Ltd., Shanghai, China) was used to determine the surface color of the samples detected by measuring L-a-b values as L* (light/dark), a* (red/green), and b* (yellow/blue), and the results were expressed by hue angle. Hue angle was obtained using the equation hue angle (*h* = arctangent (b*/a*)), where 0° equals red/purple, 90° equals yellow, 180° equals bluish/green, and 270° equals blue [17]. Every sample was measured at three equidistant points, and three samples selected randomly from each package (three package were randomly chosen from the each density active package) were analyzed [18].

### 2.6. Soluble Solid Concentration (SSC) and Titratable Acidity (TA)

The sample was ground in a mortar and squeezed by hand to obtain juice. Measurement of SSC from fruit juices using a Digital Refractometer (MZB 92, Shanghai Miqingke Industrial Testing Co., Ltd., Shanghai, China). Titratable acidity of strawberry was determined according to the international standard ISO 750-1981. A certain weight of strawberry juice was diluted 100 times with distilled water and filtered to remove the pulp. Two grams of homogenate equal a one gram sample. The samples were heated in a 70–80 °C water bath for 30 min while shaking and cooled to room temperature. Phenolphthalein was used as an indicator. The acidity was measured using a 526 WWW pH meter (Merck, Barcelona, Spain) with a glass electrode. The acidity was measured by titration with 0.1 mol·L^−1^ NaOH to pH 8.1 and expressed as a percentage of citric acid.

### 2.7. Determination of Vitamin C

Vitamin C content in strawberries was determined by Spectrophotometric method. An appropriate amount of water was added to the sample to make the homogenate, which was centrifuged at 10,000× *g* for 10 min and then the supernatant was removed. The dilution times of the sample fluid depend on VC content and sample fluid color. In this study, 1:10 was used as the solid-to-liquid ratio to make the homogenate, as the color of the homogenate was too dark to be determined by spectrophotometry (UV-1800, Mapada Instruments Co., Ltd., Shanghai, China) [19]. Then, the homogenate was diluted 10 times with distilled water. Distilled water was used as a reference, and a series of VC standard solutions were prepared. The absorption spectrum curve of VC was plotted in the range of 220–320 nm to determine the maximum absorption wavelength. The absorbance was finally measured at 267 nm to draw a standard curve and then the vitamin C content of the sample was calculated from the standard curve [18].

### 2.8. Total Phenolics Content

Total phenolics content in strawberries were determined by the Folin–Ciocalteu method [20]. Briefly, the samples (10 g) were mixed with 10-fold 80% cold methanol (100 mL) at room temperature for 30 min. During this time, the strawberries were thoroughly ground in a methanol (100 mL) mortar and pestle. Then, the homogenate was centrifuged at 10,000× *g* for 20 min and filtered. The supernatant was taken and the residue was extracted twice. The supernatants were combined. The crude extract (1 mL) was mixed with 10-fold diluted 2 N Folin–Ciocalteu reagent (4.0 mL). The mixture was kept at room temperature for 5 min and then 4 mL of Na_2_CO_3_ (7.5% *w*/*v*) was added. Before the mixture was incubated for 1 h at room temperature, it was allowed to react in a vortex mixer. The Absorbance was measured by spectrophotometer at 765 nm. The result was expressed as mg·GAE/100 g FW. The amount of total phenolics in the strawberries was calculated using a gallic acid calibration curve.

### 2.9. 1-Diphenyl-2-Picrylhydrazyl (DPPH) Determination

DPPH free radical scavenging activity of sample extracts was determined following the method described by Cao et al. [21] to assess antioxidant capacity. Crude sample extraction method was the same as total phenol extraction method. Exactly 0.0394 g DPPH was weighed and diluted with 100 mL of 95% methanol to obtain 1 mmol/L DPPH liquor, which was diluted 12 times with methanol to form 0.12 mmol/L DPPH reaction solutions. Then, 0.1 mL sample solution and 2.9 mL DPPH reaction solution were put together in a 5 mL centrifuge tube. The mixture was shaken for 30 min in a dark place at room temperature (Absorbance_sample_). The absorbance was measured at a wavelength of 517 nm using spectrophotometry. The absorbance of 2.9 mL DPPH reaction solution and 0.1 mL methanol mixture was measured (*A*_0_). The absorbance of water in the DPPH reaction mixture was measured simultaneously under the same condition (*A*_1_). *A*_0_ − *A*_1__⁼_ Absorbance_control_. All assays were performed in triplicate. The result was calculated according to Equation (2):

(2) $$\mathrm{DPPH}\;\mathrm{radical}\;\mathrm{scavenging}\;\mathrm{activity}(\%)=(1\;-\frac{{\mathrm{Absorbance}}_{\mathrm{sample}}}{{\mathrm{Absorbance}}_{\mathrm{control}}})\;\times \;100\%$$

### 2.10. Pyrogallol Peroxidase Assay

The pyrogallol peroxidase (POD) activity was measured according to Chen et al. [22] with some modifications. The fresh fruits were crushed into coarse pieces; 5-g samples were homogenized for 3 min with 20 mL of pre-cooled sodium phosphate buffer (0.1 mol/L, pH 6.8) and 2 wt % of PVP in a cooled mortar and pestle. The homogenate was collected into the centrifuge tube and centrifuged (TGL-16M, Xiangyi Centrifuge instruments Co., Ltd., Shanghai, China) at 10,000× *g* for 20 min. During centrifugation, the homogenate was kept at 4 ± 1 °C. Then, the supernatant was used as a crude enzyme solution. The reaction mixture consisted of 0.5 mL of crude enzyme solution, 3 mL of 25 mM guaiacol, and 200 μL of 0.5 M hydrogen peroxide (30%). Distilled water was used as a reference. The POD value was measured at 470 nm. The measurement was started after 15 s of reaction and then repeated three times every minute. One unit of enzyme activity was defined as an increase in absorbance of 0.001/min. The result was expressed as U per gram of fresh fruit (U·g^−1^ FW).

### 2.11. Sensory Evaluation of Strawberries

The quality assessment consisted of ten trained reviewers from Yunnan Institute of Food Safety, Kunming University of Science and Technology, Kunming, China. A nine-point hedonic scale based on color, texture, odor, and overall acceptability of samples was used to differentiate changes in sample quality, where 1 = inedible, 3 = poor, 5 = fair, 7 = very good, and 9 = excellent [23].

### 2.12. Microbiological Analysis

The total bacterial count of the sample was evaluated according to the plate count method. Briefly, 25 g of samples was aseptically transferred to 225 mL of a 0.85% (*w*/*v*) sterile physiological saline solution and homogenized. Serial decimal dilutions were prepared in sterile peptone water and poured onto plate count agar (PCA) plates. The total bacterial count was incubated on plate agar (Oxoid, London, UK) for 48 h at 30 °C. All counts were the average of the samples and expressed as log cfu/mL.

### 2.13. Statistical Analysis

The result was represented as means ± standard deviations and analyzed by analysis of variance (ANOVA) using SPSS (version 19.0, SPSS Inc., Chicago, IL, USA). Duncan’s multiple-range test was used to determine significant differences at 95% confidence level.

## 3. Results and Discussion

### 3.1. Weight Loss

**Figure 1:**
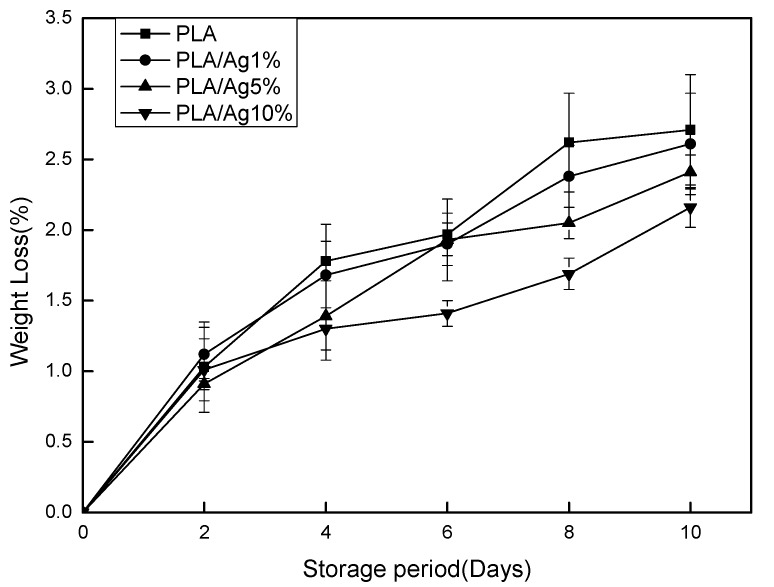
Effect of different concentration of nano-Ag active packages on weight loss of strawberry stored at 4 ± 1 °C for 10 days. Data are presented as mean ± standard deviation.

The weight loss value of strawberry samples during the preservation period is shown in Figure 1. During the preservation period, the weight loss of all packaged samples gradually increased, which might be related to the continuous loss of water from the strawberries to the surrounding environment. As shown in Figure 1, after two days of storage, the mass loss of the strawberry samples packed with PLA/Ag10%, PLA/Ag5%, and PLA/Ag1% film was significantly (*p* < 0.05) lower than that of the PLA films. This was because a certain amount of nano-Ag was incorporated in PLA matrix. Nano-Ag has an outstanding antimicrobial property. It can attach to the cell membranes and penetrate into bacteria, block the bacterial respiratory chain, and eventually kill the bacteria on the surface of the attached material. Thus, higher concentration nano-Ag package has higher antimicrobial property. The fruit packed in the high concentration nano-Ag package was less prone to spoil and deteriorate [24]. The weight loss of the fruit packaged by different concentration film was presented as: PLA film > PLA/Ag1% film > PLA/Ag5% film > PLA/Ag10% film.

### 3.2. Firmness

**Figure 2:**
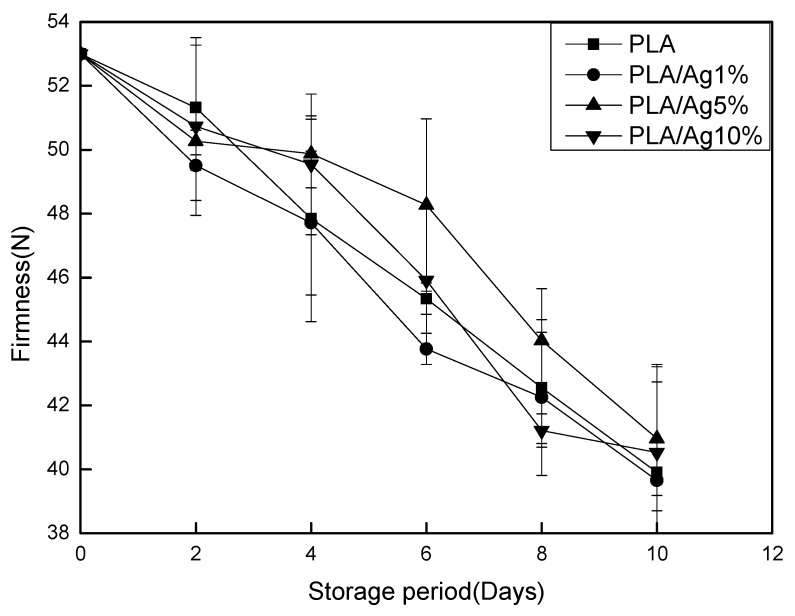
Effect of different concentration of nano-Ag active packages on firmness of strawberry stored at 4 ± 1 °C for 10 days. Data are presented as mean ± standard deviation.

Firmness is one of the important indicators for strawberries. The change trend of the tissue firmness of strawberry samples packaged in different packaging films is shown in Figure 2. During the 4 ± 1 °C preservation period, the firmness value of all samples showed a downward trend. After 10 days of storage, the PLA/Ag5% film package had the highest firmness value (40.96 g) and the PLA film package had the lowest firmness value (39.65 g). This might be because the water vapor permeability of nano-mixed membranes was higher than that of other membranes and nano-Ag has certain antibacterial properties. Low-permeability packaging films could increase the relative humidity inside the package to accelerate the softening of the strawberries. Studies have shown that, during the storage period, strawberry tissue becomes soft due to metabolic changes and moisture loss of the enzyme, which in turn reduces the firmness of the strawberry [25].

### 3.3. SSC and TA

**Figure 3:**
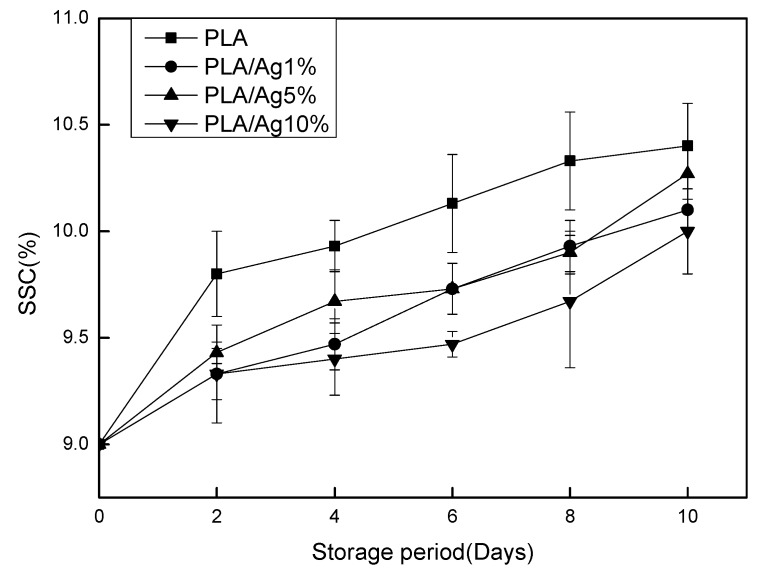
Effect of different concentration of nano-Ag active packages on soluble solid content of strawberry stored at 4 ± 1 °C for 10 days. Data are presented as mean ± standard deviation.

The amount of SSC in strawberries could be used to assess the ripeness of strawberries and fully mature strawberries contain the most soluble solids [26]. In Figure 3, longer storage periods leads to greater soluble solids content in the four groups of strawberries. During the first two days of storage, there was no significant (*p* > 0.05) difference in SSC between PLA/Ag1% and PLA/Ag5% group. The increase in SSC value in the four groups of strawberries was because the starch in the strawberries continuously converted to soluble sugars as the storage period increased. PLA/Ag5% and PLA/Ag10% film had better inhibition of strawberry respiration and inhibition of metabolic enzyme activity than PLA film. Therefore, the soluble solids growth of strawberry coated with PLA/Ag5% and PLA/Ag10% film was slower.

**Figure 4:**
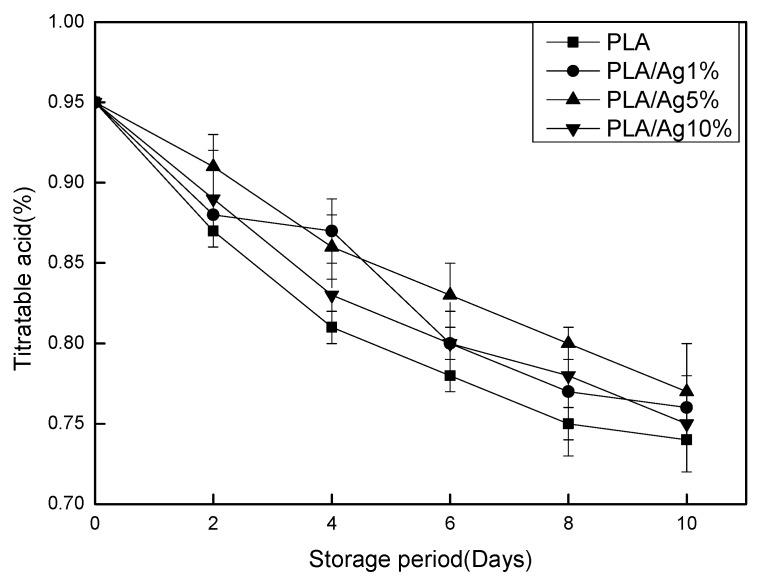
Effect of different concentration of nano-Ag active packages on titratable acid of strawberry stored at 4 ± 1 °C for 10 days. Data are presented as mean ± standard deviation.

The amount of TA in strawberries is closely related to their flavor, and the acid content decreases as the respiratory metabolism of strawberries increases. In Figure 4, the TA content in the four groups of strawberries decreased continuously with the extension of the storage period. In the PLA group, TA value rapidly decreased most in the strawberry, followed by the PLA/Ag1% group, and was slowest in the PLA/Ag5% and PLA/Ag10% groups. In the whole process of storage, the content of TA of nano-active packaging film is higher than that of pure polylactic acid packaging film, indicating that nano-modified film is conducive to delaying the decrease of TA content of fruits during storage. This is consistent with the findings of Li et al. [27]. The experimental results showed that the nano-composite membrane could inhibit the respiration of strawberry and slow down the consumption of acid in the physiological metabolic activities of strawberry, thus effectively slowing down the downward trend of titratable acid and extending the shelf life of the strawberries.

### 3.4. Surface Color

**Figure 5:**
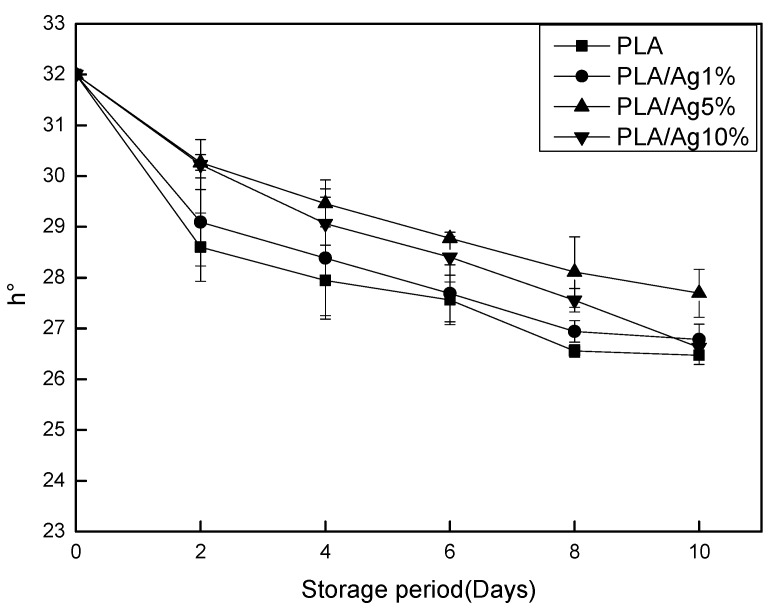
Effect of different concentration of nano-Ag active packages on the hue angle of strawberry stored at 4 ± 1 °C for 10 days. Data are presented as mean ± standard deviation.

During storage, discoloration mainly occurs on the surface of the strawberry and is mainly manifested as darkening or spoilage. This greatly reduces the nutritional value and sensory characteristics of strawberry itself, and seriously affects consumers’ desire to purchase products. Among them, the enzymatic browning of the reaction of polyphenol compounds with oxygen catalyzed by the endogenous polyphenol oxidase (PPO) [28] in strawberry is a major problem. Hue angle was used to characterize the color change of strawberry surface. In Figure 5, the degree of coloration of the samples in each group of packaging films during storage gradually decreased. On Day 10 of storage, the hue angles of PLA, PLA/Ag1%, PLA/Ag5%, and PLA/Ag10% decreased by 18.31%, 16.31%, 13.47%, and 16.78%, respectively, when compared with the initial value. The color difference value of the fruit in the PLA active packaging film, in which the amount of nano-Ag was added at 5%, was lower than the initial value. The value of the hue angle was always higher than other groups, and the difference was significant (*p* < 0.05). The concentration of the active packaging film showed better protection of strawberry surface color than other concentrations. However, the result showed that, although the different processing and storage methods could improve the decline of the surface color of the strawberry, the change of the surface color of the strawberry could not be used as an indicator to effectively reflect the strawberry fruit quality [29].

### 3.5. Determination of Vitamin C

**Figure 6:**
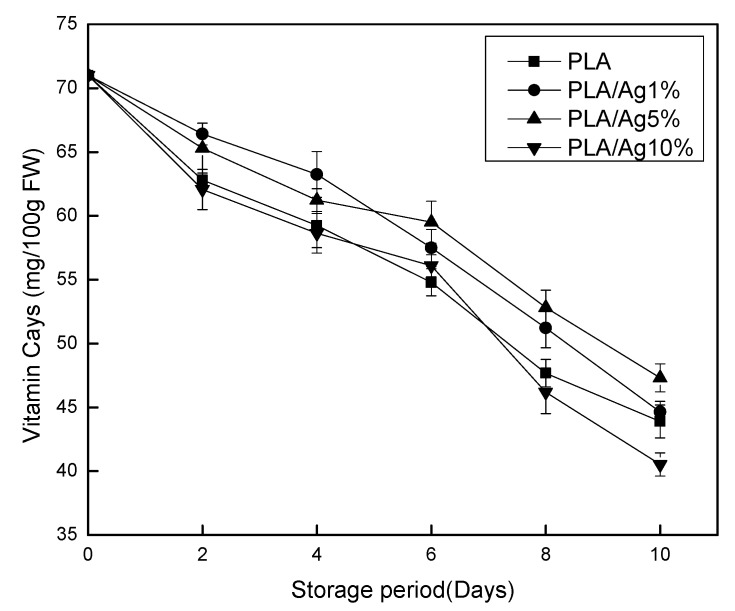
Effect of different concentration of nano-Ag active packages on the vitamin C level strawberry stored at 4 ± 1 °C for 10 days. Data are presented as mean ± standard deviation.

The antioxidant properties of vitamin C play an important role in the metabolism of plants and animals [30]. Since the human body cannot synthesize vitamin C, it can only meet the needs of the human body through diet or supplementary nutrients, and a very healthy way to supplement vitamin C is eating fruit. Thus, vitamin C plays a vital role in the preservation of food, especially in vegetable and fruits during the storage. As Mcerlain and Marson mentioned, improper preservation methods can accelerate the loss of vitamins in food [31]. Therefore, the change of VC content in strawberry in different content of nano-Ag packaging film can verify whether nano-Ag film package has a good preservation effect. Changes in vitamin C content of strawberries during storage are shown in Figure 6, in which the content of vitamin C decreases with time for all the samples, which might be attributed to its oxidation through OH radicals in the strawberries [32]. It could be seen in the figure that vitamin C content decreased linearly after storage. With the prolongation of storage time, the strawberry in all active packaging films gradually showed significant (*p* < 0.05) difference in vitamin C content compared with pure polylactic acid from Day 4 until the end of storage days. Among them, PLA/Ag1% is significantly (*p* < 0.05) different from PLA during the whole storage process. Over time, the vitamin C content of strawberries in 5% nano-Ag active packages was higher than other active packaging film concentrations during storage. On Day 10 of storage, the content of ascorbic acid in the pure PLA film and nano-Ag-added active packaging had a significant (*p* < 0.05) difference from the initial value, indicating that the nano-Ag active film can effectively reduce the loss of ascorbic acid in strawberry. This might be due to the addition of nano-Ag, changes in membrane permeability [18], inhibition of respiration, and delay in occurrence of ascorbic acid oxidation reaction. The ascorbic acid content of strawberry in the packaging of nano-Ag films with a low to high concentration decreased by 38.18%, 37.13%, 33.38%, and 42.93%, respectively, compared with the initial values.

### 3.6. Pyrogallol Peroxidase (POD)

**Figure 7:**
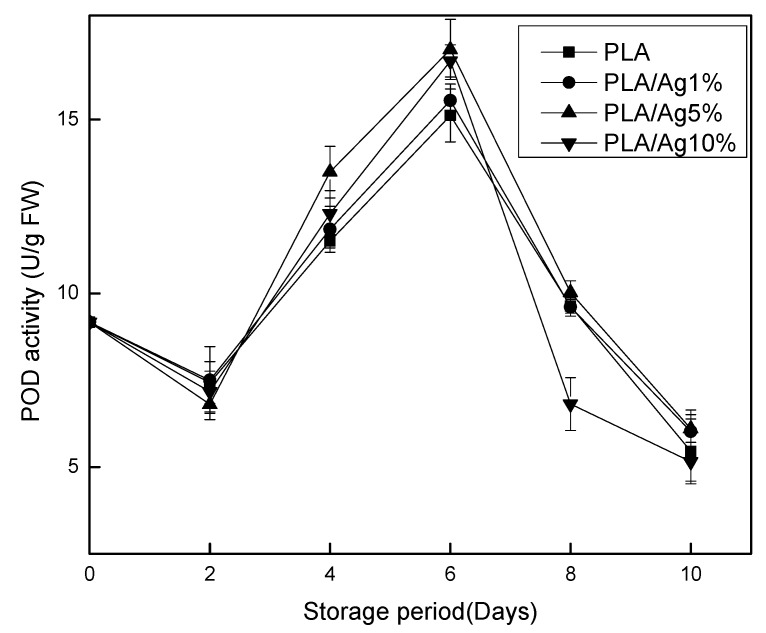
Effect of different concentration of nano-Ag active packages on the activity of POD of strawberry stored at 4 ± 1 °C for 10 days. Data are presented as mean ± standard deviation.

Enzymatic browning caused by oxidation of phenolic compounds is one of the most important causes of fruit color deterioration. POD is one of the oxidoreductases involved in enzymatic browning [33]. It might cause not only spoilage of the appearance of the fruit but also the weight loss. In Figure 7, there was no significant (*p* < 0.05) difference in pod content in each packaging film during the first two days of storage. The pod activity in all active packaging film packages gradually increased until the activity peaks on Day 6. The maximum pod activity values of PLA, PLA/Ag1%, PLA/Ag5% and PLA/Ag10% were 15.12, 15.55, 17.02 and 16.69 U/g FW, respectively. PLA and all active packaging films are significantly (*p* < 0.05) different. PLA/Ag5% film was higher than that of other concentrations of nano-active film and pure PLA film samples. Liu et al. [34] found that POD activity of mushrooms increased first and then decreased during storage. Moreover, the POD activity of mushrooms was always higher than that of the control group, so it was beneficial to prolong the storage period of mushrooms, which was consistent with the results of this study. This could be due to the antibacterial properties of the nanoparticles in the film, which could delay fruit senescence. The high activity of POD indicated that the sample had a low degree of tissue aging and that the fruits and vegetables were fresher. After Day 6, the activity gradually decreased. On Day 8, PLA10 was significantly lower than all packaging films. Then, there was no significant (*p* < 0.05) difference between the packaging films over time. The POD enzyme activity increased rapidly from the beginning of storage to Day 6, possibly due to the POD-induced enzyme action. When the fruit was adversely affected by the outside, the enzyme activity increased; and the higher the cellular activity, the greater the increased [35]. As the POD activity declined, the quality of the fruit began to deteriorate. Studies have shown that POD may be involved in the production of ethylene in fruits and can be used as an indicator of fruit ripening and senescence [36]. This might regulate the maturation of the strawberry and ultimately improve the sensory quality of the fruit in the nano-active packaging film. Considering fruit preservation effect and material cost savings, from Day 0 to Day 6, PLA/Ag5% film is better.

### 3.7. Total Phenolics Content

**Figure 8:**
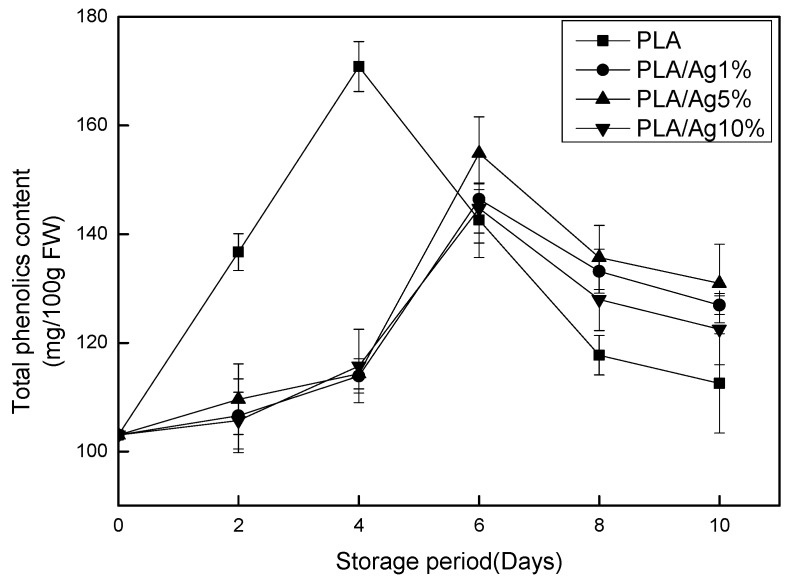
Effect of different concentration of nano-Ag active packages on total phenolics of strawberry stored at 4 ± 1 °C for 10 days. Data are presented as mean ± standard deviation.

Total phenols are very important for the quality of strawberries, not only because of their contribution to the taste, but also polyphenols and anthocyanins affect the appearance of pigments in fruits and vegetables [37]. Phenols have anti-aging, anti-cancer, anti-inflammatory and anti-oxidation biological functions, making total phenols an important indicator to assess the fruit and vegetable preservation effect [38]. Changes in the total phenolic content of the sample during storage are shown in Figure 8. In Figure 8, the content of total phenols encapsulated by various contents of nano-Ag increased at first and then decreased. The total phenolic content of the pure PLA packaging film increased rapidly after storage began and peaked on Day 4. The content of total phenols in the nano-Ag packaging films with different contents rose slowly and peaked on Day 6. The peak value was significantly (*p* < 0.05) lower than the peak value of total phenolics in the pure PLA packaging film. From Day 6 to Day 10, the total phenolic content of strawberry in pure PLA packaging film and nano-Ag active packaging film showed a decreasing trend, but the total phenol content of nano-Ag active packaging film was higher than that of pure nano-Ag packaging film. The content is maintained at a high level and the downward trend tends to be gentle. Yang et al. showed that this might be due to the fact that nano-packaging inhibits the accumulation of anthocyanin in fruits [39]. The rapid accumulation of total phenols in the first four days might be due to the accumulation of other phenolic substances. Shin et al. found that changes in the total phenolic content of strawberries during storage are affected by the maturity at harvest [40]. The total phenolic content of fully ripened strawberries will continue to decrease during storage. The experimental samples maturation were all 70–80% but not fully matured. Therefore, the phenols gradually accumulated during the early storage and led to increased levels. Studies have also shown that higher concentrations of oxygen in the environment will accelerate the accumulation of total phenols in fruits [41]. The nano-Ag particles added to the PLA film change the oxygen and carbon dioxide transmission rate, which in turn reduces the oxygen content in the package [39]. Therefore, the nano-Ag active packaging film has lower total phenol content than the pure PLA packaging film, which can effectively delay the decay of the total phenol content. In addition, the total phenol content in the active packaging film does not increase with the increase of nano-Ag content in the active packaging film. In the low concentration range of the active film, the Ag concentration of 5% has higher total phenol content than other concentrations of the nano-Ag active packaging film. The overall trend of nano-Ag films with a concentration of 10% is higher than that of pure PLA films lower than that of low-concentration nano-Ag films. This might be because, as the nanoparticles are added, the nanoparticles are aggregated, the voids of the membrane are enlarged, and the respiration continuously causes the loss of phenolic content.

### 3.8. 1-Diphenyl-2-Picrylhydrazyl (DPPH)

**Figure 9:**
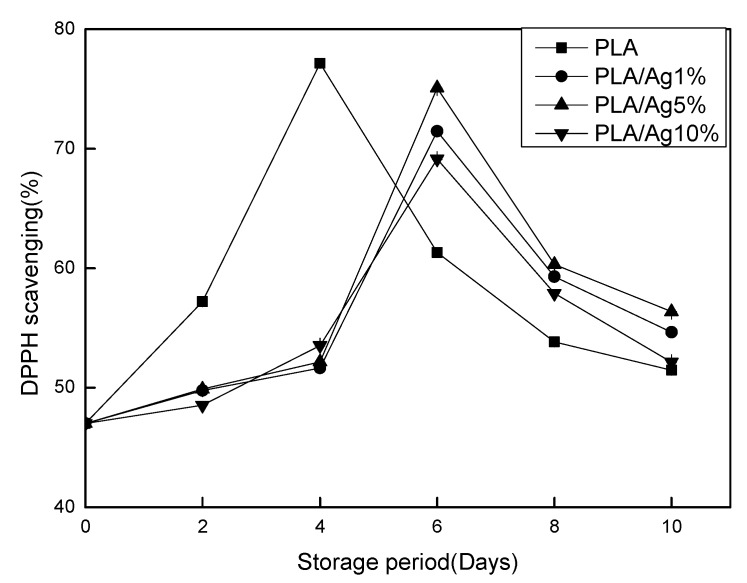
Effect of different concentration of nano-Ag active packages on antioxidant capacity measured by DPPH. Data are presented as mean ± standard deviation.

Antioxidant ability is one of the important indicators for evaluating fruits and vegetables. There are many methods to determine the antioxidant capacity of samples, such as ferric reduction antioxidant (FRAP) method, oxygen radical absorbance capacity (ORAC) method for measuring antioxidant capacity, and so on. However, the DPPH method has been widely used in the determination of the antioxidant capacity of fruits and vegetables and their extracts due to their sensitivity, rapidity, reliability, and simple method [42]. In Figure 9, the DPPH radical scavenging rates of the packaging films of strawberry during the whole storage period showed a trend of increasing first and then decreasing. On Day 4, the pure PLA film reached its peak first and then decreased rapidly. On Day 6, the DPPH radical scavenging rate of each group of nano-Ag active packaging films also peaked and then decreased and maintained at a relatively high level. There was a significant (*p* < 0.05) difference between the numerical value and the pure PLA film. Li and coworkers’ [43] research results are consistent with the results of this study.

### 3.9. Microbiological Analysis

**Figure 10:**
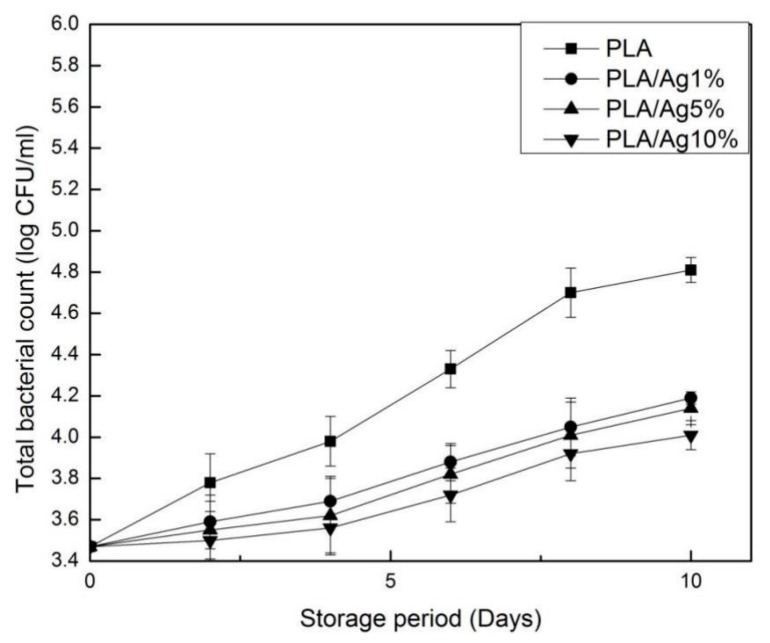
Effect of different concentration of nano-Ag active packages on the account of total bacterial. Data are presented as mean ± standard deviation.

Total bacterial is one of the indicators for judging food hygiene. The change in the total number of bacteria during storage is shown in Figure 10. The total number of bacteria gradually increased throughout the storage period. There was no significant (*p* > 0.05) difference in the total number of bacteria between the PLA/Ag1% and PLA/Ag5% groups during the entire storage period. The total number of bacteria in the PLA/Ag10% wrapped strawberry samples was significantly (*p* > 0.05) lower than that in PLA. This may be because the incorporation of nano-Ag into PLA can effectively inhibit the growth of fruit microorganisms. This may be related to the antibacterial mechanism of nano-Ag. The antibacterial mechanism of nano-Ag is related to membrane damage caused by free radicals derived from the surface of nano-Ag [44]. Fan et al. [45] found that nano active film is better than PLA film for food preservation. Li and coworkers’ [46] study shows that the higher content of ZnO composite film with 3 wt % content has the best preservation performance compared with 0 wt % and 1 wt %.The experimental results are consistent with the above studies.

### 3.10. Sensory Evaluation of Strawberries

The sensory evaluation of the strawberry was performed on a nine-point scale, and the odor, color, texture, and overall acceptability were evaluated. Table 1 lists the sensory scores for strawberries, with the values for all groups decreasing with increasing storage time. There was no significant (*p* > 0.05) difference in sensory scores of all packaged samples on Day 2. However, after eight days of storage, the odor and texture of the strawberry samples packed with PLA/Ag1%, PLA/Ag5%, and PLA/Ag10% were not affected. The overall acceptability score was significantly higher than that of the PLA film. At the end of the storage time, the overall acceptability scores of samples packaged with PLA/Ag1%, PLA/Ag5%, and PLA/Ag10% were still higher than 5, and the sample maintained proper quality characteristics considering the sensory parameters such as odor, color, and texture. After 10 days of storage, the overall acceptability of the sample packed with PLA/Ag5% film was highest, and it was not significantly (*p* > 0.05) different from that of PLA/Ag%1 and PLA/Ag10% films. Six points are the criteria for good product quality and marketability. The shelf life of strawberry samples packed with PLA, PLA/Ag1%, PLA/Ag%5 and PLA/Ag10% films was four, six, eight and six days, respectively. The results showed that PLA/Ag film can improve the quality of strawberry during refrigerated storage. The main reason is that the nanofiber membrane has a small pore size and a high porosity. It can prevent gas and water from passing through and can block foreign pollutants from infecting the sample. It also improves the hydrophilicity of the membrane and prevents condensation of water vapor on the inner surface of the membrane [47].

## 4. Conclusions

Compared with pure PLA film, nano-Ag active film could effectively reduce the weight loss rate of strawberries during storage, and delay the drop in hardness, soluble solids, and titratable acid content. High levels of ascorbic acid and total phenols were maintained during the late storage period. The strawberries had a higher antioxidant capacity in the later period of storage, thus delaying the aging of the fruit and providing a better preservation effect. Therefore, the nano-Ag active packaging film has a better preservation effect, and PLA/Ag5% film is slightly better than other films.

## Figures and Tables

**Table 1:** Sensory evaluation of strawberries stored for 10 days at 4 ± 1 °C in different concentration of nano-Ag active packages.

Treatment	Odor	Color	Texture	Overall Acceptability
Day 0	9	9	9	9
Day 2				
PLA	8.18 ± 0.03 ^ab^	7.87 ± 0.32 ^a^	8.07 ± 0.17 ^a^	7.94 ± 0.02 ^a^
PLA/Ag1%	8.05 ± 0.18 ^a^	8.06 ± 0.32 ^a^	8.22 ± 0.04 ^ab^	8.05 ± 0.59 ^a^
PLA/Ag5%	8.29 ± 0.07 ^b^	8.08 ± 0.05 ^a^	8.28 ± 0.07 ^b^	8.13 ± 0.11 ^a^
PLA/Ag10%	8.22 ± 0.04 ^ab^	8.07 ± 0.15 ^a^	8.19 ± 0.04 ^ab^	8.04 ± 0.02 ^a^
Day 4				
PLA	7.19 ± 0.04 ^a^	7.05 ± 0.39 ^a^	6.98 ± 0.14 ^a^	6.89 ± 0.27 ^a^
PLA/Ag1%	7.37 ± 0.18 ^a^	6.85 ± 0.22 ^a^	7.05 ± 0.18 ^a^	7.07 ± 0.01 ^ab^
PLA/Ag5%	7.37 ± 0.13 ^a^	7.51 ± 0.44 ^a^	7.18 ± 0.01 ^a^	7.2 ± 0.08 ^b^
PLA/Ag10%	7.24 ± 0.02 ^a^	7.19 ± 0.21 ^a^	7.12 ± 0.08 ^a^	7.12 ± 0.06 ^ab^
Day 6				
PLA	6.68 ± 0.06 ^ab^	6.27 ± 0.21 ^a^	6.45 ± 0.05 ^a^	6.03 ± 0.4 ^a^
PLA/Ag1%	6.67 ± 0.02 ^ab^	6.31 ± 0.17 ^a^	6.49 ± 0.01 ^ab^	6.52 ± 0.02 ^b^
PLA/Ag5%	6.76 ± 0.09 ^b^	6.63 ± 0.02 ^b^	6.55 ± 0.04 ^b^	6.71 ± 0.05 ^b^
PLA/Ag10%	6.59 ± 0.1 ^a^	6.52 ± 0.15 ^ab^	6.46 ± 0.04 ^a^	6.4 ± 0.08 ^ab^
Day 8				
PLA	6.22 ± 0.02 ^ab^	5.17 ± 0.14 ^a^	5.99 ± 0.27 ^a^	4.78 ± 0.06 ^a^
PLA/Ag1%	6.13 ± 0.16 ^a^	5.38 ± 0.21 ^a^	6.1 ± 0.07 ^a^	5.9 ± 0.04 ^ab^
PLA/Ag5%	6.29 ± 0.05 ^b^	5.43 ± 0.21 ^a^	6.12 ± 0.35 ^a^	6.3 ± 0.56 ^b^
PLA/Ag10%	6.16 ± 0.03 ^ab^	5.34 ± 0.06 ^a^	6.08 ± 0.05 ^a^	4.82 ± 1.02 ^a^
Day 10				
PLA	5.71 ± 0.23 ^b^	5.01 ± 0.55 ^a^	5.45 ± 0.12 ^ab^	4.23 ± 0.57 ^a^
PLA/Ag1%	5.05 ± 0.08 ^a^	5.03 ± 0.09 ^a^	5.36 ± 0.12 ^a^	5.36 ± 0.24 ^b^
PLA/Ag5%	5.66 ± 0.32 ^ab^	5.14 ± 0.25 ^a^	5.6 ± 0.03 ^b^	5.73 ± 0.04 ^b^
PLA/Ag10%	5.13 ± 0.49 ^ab^	4.99 ± 0.02 ^a^	5.46 ± 0.06 ^ab^	5.35 ± 0.15 ^b^

a–b Values followed by different letters in the same column were significantly different (*p* < 0.05), where a is the lowest value.
